# Novel infrared puffing: Effect on physicochemical attributes of puffed rice (*Oryza sativa* L.)

**DOI:** 10.1002/fsn3.3022

**Published:** 2022-08-09

**Authors:** Mahdi Shavandi, Majid Javanmard, Alireza Basiri

**Affiliations:** ^1^ Food Technologies Group, Department of chemical Engineering Iranian Research Organization for Science & Technology (IROST) Tehran Iran

**Keywords:** cereal grains, infrared expansion, infrared puffing, rice

## Abstract

The effect of novel infrared (IR) puffing and various IR powers (350, 450, and 550 Watts [W]) at various distances (10, 20, and 30 cm) on physicochemical characteristics of puffed rice (puffing properties, color, total phenolic content [TPC], antioxidant activity, peroxide value, and morphology) was investigated. By reducing the distance and increasing the IR power, the volume puffing was significantly increased (*p* < .05), and bulk density was significantly decreased (*p* < .05) but there was no significant difference in the length/breadth ratio. The IR puffing effect on color, the TPC, antioxidant activity, and food compounds' analysis through Fourier transform infrared (FTIR) spectra were significant (*p* < .05) during IR puffing. The scanning electron microscopy (SEM) images showed that by increasing the IR power and decreasing the sample distance from the IR source, the size of protrusions was increased (the volume of the protrusions). The maximum increase in the protrusions size was observed in 10 cm distance and 550 W power of IR. This is the first report on the IR puffing of rice and according to the results, the IR puffing technology has a high efficiency at the rice puffing.

## Graphical abstract

The effect of novel infrared (IR) puffing, various IR power (350, 450 and 550 watts (W)) at various distances (10, 20 and 30 cm) on physicochemical characteristics of puffed rice (puffing properties, color, total phenolic content (TPC), antioxidant activity, peroxide value and morphology) was investigated.

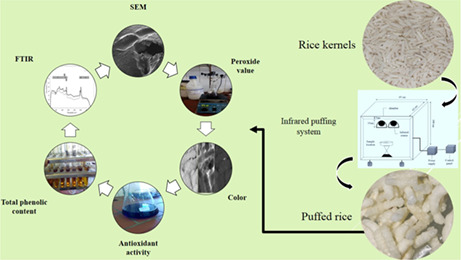

## 1 INTRODUCTION

Rice (*Oryza sativa*) is consumed as a main dish by about half the people of the world (Valipour, 2017). Rice has health properties, and contributes one part of three parts of the recommended range of protein intake in a day, about 60%–70% of the daily total calories requirement and many other useful nutrients (Joshi et al., 2014; Maisont & Narkrugsa, 2010). Rice is a main dish that is consumed in the form of snack and expanded form (puffed/popped), ready‐to‐eat food, extruded, and flaked products (Kamaraddi & Prakash, 2015).

Rice is generally used for the purpose of puffing process due to its nutritional profile, texture, and taste and puffed rice is a popular expanded cereal. Generally, puffed rice is prepared by parboiling the rice under the specified conditions followed by high temperature short time (HTST) treatment in sand or hot air (Chinnaswamy & Bhattacharya, 1983; Murugesan & Bhattacharya, 1991).

The properties of its expansion are related to consumer acceptation and sensory properties. Changes in functional properties and favorable sensory properties of expanded products by the puffing/popping process cause to increase consumers' demand (Dharmaraj et al., 2012). Rice puffing changes the nutritional profile and physical properties of the grain due to thermal treatments during the processes. For example, the rice parboiling is a hydrothermal process, which modifies the processing and qualitative behavior (Dutta & Mahanta, 2012).

Starch granules in rice kernels are retrograded and gelatinized during the parboiling process and their quality attributes change. The parboiling process basically brings about the properties' change in the rice, which leads to expansion during the puffing process. On puffing, structural, crystallinity, conformational, and appreciable physical changes occur in rice grains, leading to a change in the texture and morphology (Shih et al., 2007). The grains heating using hot air is a high‐energy consumption process. The waste exhaust heat is high and heat utilization factor is also high. It was suggested that methods based on radiation such as infrared (IR) and microwave radiation are more economical and convenient (Devi & Das, 2018).

Fluidized bed (Llopart & Drago, 2016), aluminum popcorn popper (Hoseney et al., 1983), atmospheric radio frequency plasma (Puangjinda et al., 2016), microwave heating (Cañizares et al., 2020), and iron pan containing sand (Mir et al., 2016) have been used for puffing/popping of cereals. The effect of butter (1% to 13%), sodium chloride (0.5% to 2.5%), sodium bicarbonate (0.0% to 0.8%), and vegetable oil (1% to 13%) on popcorn properties was investigated (Singh & Singh, 1999). In a study, the effects of moisture content (8% to 16%) and expansion (with oil and without oil) of white popcorn on the sensory properties were investigated. It was reported that 11.39% (without oil) and 10.21% (with oil) were optimum moisture content for expansion yield (Cañizares et al., 2020).

Infrared (IR) radiation is highly energy efficient and environment‐friendly compared to conventional heating. The IR efficiency is determined by food safety, heating homogeneity, improved product quality, high heat transfer, low total energy consumption, and low processing (heating) time (Aboud et al., 2019). The IR radiation was used in many foods processing methods, such as drying, heating, microbiological decontamination, peeling, cooking, roasting, and baking (Aboud et al., 2019; Shavandi, Sadeghi, & Sarani, 2020). IR between ultraviolet (UV) and microwave wavelength is an electromagnetic spectrum part (1 mm to 0.76 μm) (Krishnamurthy et al., 2008).

In this study, the main objectives were to determine the IR puffing process effect (sample distance [10, 20, and 30 cm] and IR power [350, 450, and 550 W]) on the physicochemical attributes' change of puffed rice (TPC, antioxidant activity, color, peroxide value, and morphology).

## 2 MATERIALS AND METHODS

### 2.1 Sample preparation and experimental procedure

White dehulled rice (*Oryza sativa* L.) Hashemi variety (amylose 21.02%) harvested at Astaneh Ashrafieh (Gilan Province, Iran) crop year 2020/2021 were used. The samples were cleaned, sieved, and stored. The primary moisture content of rice by oven dryer was determined (for 3 h in 140°C) (ASAE, 2000). Two kilograms of samples was adjusted to 14% of the moisture content. Ten grams of samples was placed in a Pyrex petri dish (150 mm diameter) under the IR irradiation. Three replications were performed for each treatment.

### 2.2 Puffing methods

**FIGURE 1:**
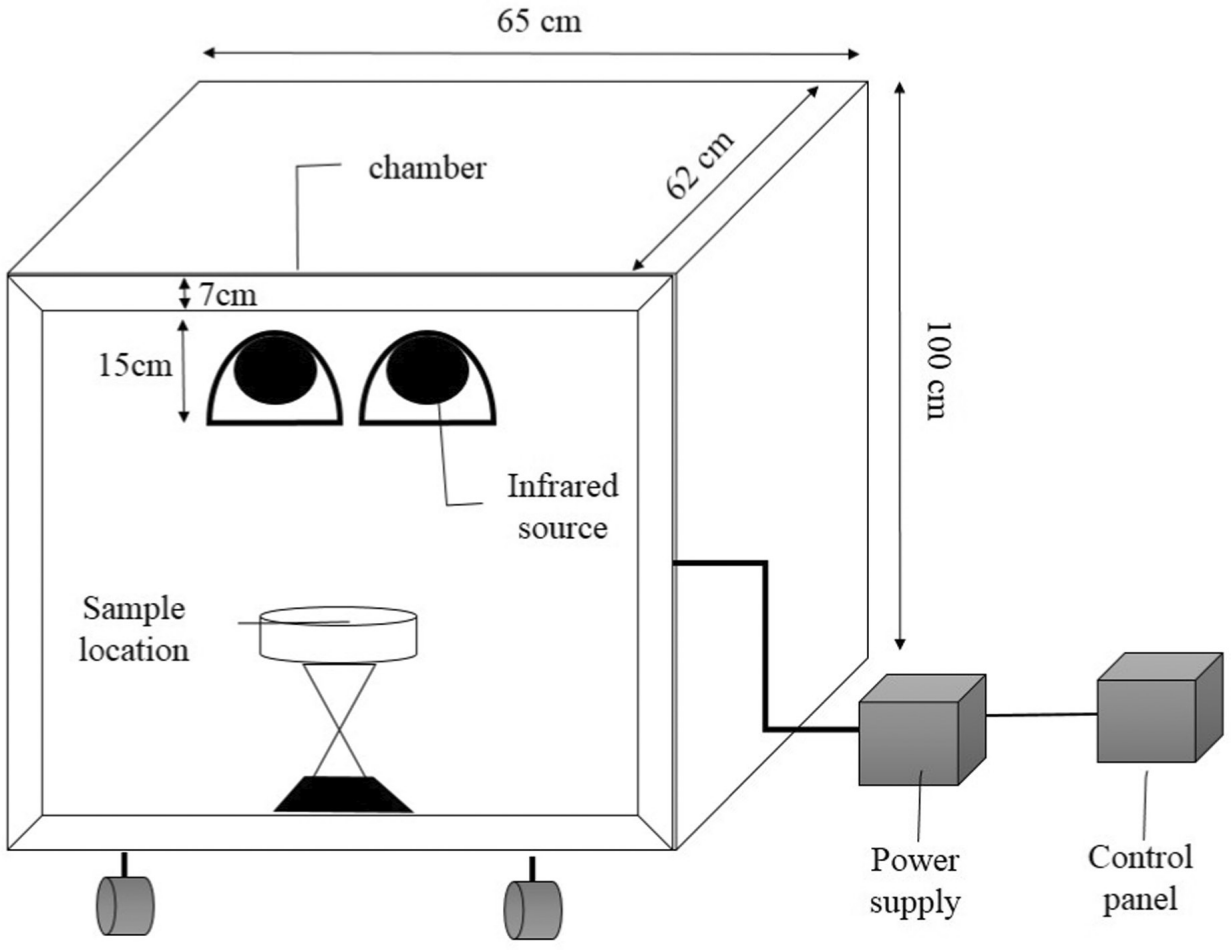
Schematic diagram of an infrared (IR) system (Shavandi et al., 2022).

The batch pilot of IR was designed and built. The IR batch pilot has a power supply, stainless steel chamber (65 × 62 × 100 cm), and 2 IR lamps (1000 Watts, 35 cm length) (Figure 1). The system was equipped with a type‐k thermocouple, a data logger (Lutron, TM‐947SD, Taipei, Taiwan), and a PC (personal computer). The effect of sample distance (10, 20, and 30 cm) and IR power (350, 450, and 550 Watts) on the puffing of puffed rice (salt 1% and moisture content 14%) was determined (Shavandi et al., 2022).

### 2.3 Determination of puffing properties

#### 2.3.1 Volume puffing

The volume puffing of puffed rice was determined in a 250 ml cylinder. The volume puffing was expressed as Equation (1):

(1) $$\text{Volume puffing ratio}=\frac{V_{f}}{V_{i}}$$

where, *v*_*i*_ = initial unpopped samples volume and *v*_*f*_ = final popped samples volume (Mishra et al., 2015).

#### 2.3.2 Bulk density

The bulk density was measured by filling puffed rice in a 250 ml cylinder using Equation (2) (Mir et al., 2016):

(2) $$\text{Bulk density}=\frac{\text{mass}\;\left(\mathrm{mg}\right)}{\text{volume}\;\left(\mathrm{ml}\right)}$$

#### 2.3.3 Length/breadth ratio

Samples at different IR treatments of puffing were randomly selected and their breadth and length were measured by using a Vernier caliper.

### 2.4 Color

The puffed rice color was determined by ImageJ software. The sample pictures were scanned and analyzed. The values of L٭ (lightness), a٭ (redness), and b٭ (yellowness) were measured (Shavandi, Kashaninejad, et al., 2020). The total color difference Equation (3), chroma Equation (4), and hue Equation (5) parameters were measured from lightness, redness, and yellowness.

(3) $$∆E=\sqrt{∆L^{2}+∆a^{2}+∆b^{2}}$$

(4) $$C=\sqrt{a^{2}+b^{2}}$$

(5) $$H=\tan^{-1}\left(b/a\right)$$

### 2.5 Antioxidant activity

#### 2.5.1 Puffed rice preparation of extract

Puffed rice was ground and passed through a sifter (150 μm). Ten grams of the samples powder was mixed by acidified methanol (100 ml) and shaking in a dark situation for 8 h at 30°C. The samples extract was filtered through Whatman filter (No. 1) and kept in a dark situation at 4°C (Mir et al., 2016).

#### 2.5.2 TPC analysis

The total phenol content (TPC) was determined through the spectroscopy method (Mir et al., 2016). The extract (0.50 ml) was added into the Folin–Ciocaltaeu reagent (2.5 ml) (1 diluted to 1:10 with distilled water) and then saturated sodium carbonate solution (10%) (2.5 ml) was added. The samples' absorbance was determined at 765 nm after 2 h having kept at room temperature in dark situation using an ultraviolet–visible (UV–Vis) spectrometer (Perkin‐Elmer Lambda 25). A gallic acid calibration curve was drawn as a reference standard. The results were reported as milligrams of gallic acid equivalents (mg GAE) per gram of samples extract.

#### 2.5.3 Antioxidant activity analysis

The antioxidant activity was measured by the designation of the free radical scavenging effect on 2,2‐diphenyl‐1‐picrylhydrazyl (DPPH) radical. As much as 0.1 ml ascorbic acid (standard) or sample extract was mixed with 1.0 ml of DPPH methanolic solution (1.0 mM) and 3.9 ml of methanol. After 30 min shaking at room temperature, the samples absorbance was determined at 517 nm using a UV–vis spectrometer (Perkin‐Elmer Lambda 25). The DPPH scavenging activity (%) was measured as Equation (6):

(6) $$\text{DPPH scavenging activity}\;\left(\%\right)=\frac{\left(A_{\text{Control}}-A_{\text{Sample}}\right)}{A_{\text{Control}}}\times 100$$

where, *A*_control_ is the control absorbance and *A*_sample_ is the extract absorbance. The concentration of the sample providing 50% inhibition (IC50) through exponential analysis of regression at Excel 2013 software (Microsoft, Inc.) was measured (Mir et al., 2016).

### 2.6 Peroxide value

#### 2.6.1 Extraction of oil from puffed rice

Puffed rice was ground, n‐hexane was added and stirred for 30 min in the dark situation at ambient temperature to prevent oxidative rancidity (Park et al., 2002).

#### 2.6.2 Peroxide value analysis

The peroxide values of the puffed rice oils were measured using the AOAC (2000) method.

### 2.7 SEM

The IR expansion effect on the puffed rice morphology was determined using scanning electron microscopy (SEM, Tescan Mira), under vacuum condition (10–4 Pa) working in 15 kV and 200x. Puffed rice as whole grain without cutting were analyzed. For high‐resolution image yield, the sample was mounted on aluminum stubs and an 8 nm layer of gold at the sample was sputtered for producing a conductive layer.

### 2.8 FTIR spectroscopy

Fourier transform infrared (FTIR) spectroscopy at the optimum samples (puffed rice) and control (raw rice) was investigated. The FTIR spectroscopy measurement was investigated using a Bruker Tensor 27 FT‐IR system (Bruker Optik, Ettlingen, Germany). The FTIR spectral range for the sample at room temperature, 400–4000 cm – 1, was determined and was registered in 2 cm^−1^ resolution.

### 2.9 Statistical analyses

Results as mean of three independent replicates and standard deviation were reported. All the data statistically using Duncan post hoc in *p* < .05 were analyzed by SAS software, version 9.3 (SAS Institute Inc.).

## 3 RESULTS AND DISCUSSION

### 3.1 Puffing attributes of puffed rice

The IR effect on the puffing attributes of puffed rice was determined. The results of the length/breadth ratio, bulk density, and volume puffing are shown in Table 1. The analysis of variance (ANOVA) showed a significant effect (*p* < .05) relative to control for volume puffing, bulk density, and the length/breadth ratio.

**TABLE 1:** Puffing properties of puffed rice through infrared (IR)

Power (W)	Distance (cm)	Length/breadth ratio	Volume puffing	Bulk density (g/cm^3^)
Raw rice		4.06 ± 0.54a	–	0.64 ± 0.04a
Conventionally puffed rice		3.29 ± 0.44b	2.22 ± 0.23a	0.30 ± 0.03ef
550	10	3.36 ± 0.59b	2.24 ± 0.31a	0.29 ± 0.03f
	20	3.05 ± 0.56b	1.85 ± 0.43c	0.35 ± 0.05e
	30	3.00 ± 0.64b	1.56 ± 0.25d	0.41 ± 0.03d
450	10	3.24 ± 0.60b	2.05 ± 0.23b	0.31 ± 0.04ef
	20	3.06 ± 0.73b	1.47 ± 0.32e	0.44 ± 0.03cd
	30	3.84 ± 0.68b	1.18 ± 0.27g	0.54 ± 0.03b
350	10	3.03 ± 0.75b	1.37 ± 0.28f	0.47 ± 0.05c
	20	3.86 ± 0.67b	1.14 ± 0.43h	0.56 ± 0.02b
	30	3.81 ± 0.63b	1.11 ± 0.31i	0.58 ± 0.04b

*Note*: Values are given as mean ± SD. *n* = 3. Different letters (a–i) within a column indicate significant difference (*p* < .05).

It was observed that with a decrease in distance and increase in the IR power, the volume puffing was significantly increased (*p* < .05), and bulk density was significantly decreased (*p* < .05) but there was no significant difference in the length/breadth ratio. Due to the change in sample size in the same direction in length and width, no significant difference was observed in the treatments. There is little difference between the different IR treatments, which may be due to differences in grain or treatment conditions.

The highest volume puffing (2.24 ± 0.31) of IR expansion of puffed rice was obtained at 10 cm distance and 550 W IR power. The lowest bulk density (0.29 ± 0.03 g/cm3) of IR puffing of puffed rice was obtained at 10 cm distance and 550 W IR power. The optimum condition for IR puffing of puffed rice was 10 cm distance and 550 W IR power.

The volume puffing can relate to the genotype, method of expansion, grains' physical attributes, and moisture content (dimensions and density) (Anne Allred‐Coyle et al., 2000; Gökmen, 2004). The maximum volume puffing occurred in the moisture content range from 15.5% to 11.0% (Anne Allred‐Coyle et al., 2000; Gökmen, 2004; Shimoni et al., 2002). The cereal grains bulk density after expansion decreased (Mariotti et al., 2006). In our previous study, similar results were observed in popcorn IR popping (Shavandi et al., 2022).

### 3.2 Color change

The color is one of the affecting parameters and important for consumers' acceptance in the food industry. Furthermore, color protection at thermal process is important and color has effect on food taste such as acceptability, preference, flavor, perception, sweetness, and saltiness (Clydesdale, 1993; Shavandi, Kashaninejad, et al., 2020).

**FIGURE 2:**
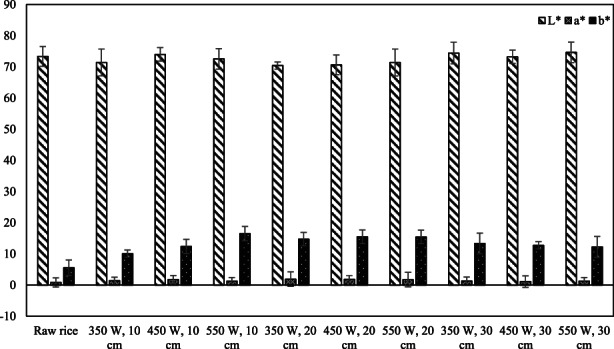
Effect of infrared (IR) on the color index in puffed rice.

Figure 2 shows the L*, a*, and b*color index and Table 2 indicates Δ*E*, hue, and chroma values of IR puffed rice and control. In the index of color, just change of b* in IR puffed rice was significant (*p* < .05). Δ*E* and chroma were significantly changed in IR puffed rice (*p* < .05). The hue index was not changed significantly in IR puffed rice (*p* > .05).

**TABLE 2:** The effect of infrared IR on Δ*E*, chroma, hue, and IC50 in puffed rice

Power (W)	Distance (cm)	Δ*E*	Chroma	Hue	IC50
Control (raw rice)		–	5.63 ± 1.41i	1.42 ± 0.35b	870.84 ± 6.21f
Ascorbic acid (the antioxidant activity standard)		–	–	–	11.09 ± 1.42k
550	10	4.95 ± 1.42f	10.17 ± 1.99h	1.43 ± 0.15ab	664.30 ± 5.35dj
	20	6.89 ± 1.01e	12.49 ± 1.67f	1.43 ± 0.23ab	901.00 ± 5.74e
	30	10.98 ± 1.70a	16.56 ± 1.8a	1.49 ± 0.19a	1150.77 ± 6.18c
450	10	9.68 ± 1.36c	14.85 ± 1.35c	1.44 ± 0.25ab	723.73 ± 7.89i
	20	10.34 ± 0.99b	15.60 ± 1.37b	1.45 ± 0.19ab	914.02 ± 4.92d
	30	10.11 ± 1.21bc	15.54 ± 2.01b	1.46 ± 0.24ab	1294.39 ± 5.88b
350	10	7.87 ± 0.89d	13.41 ± 1.62d	1.47 ± 0.34ab	725.63 ± 4.76dh
	20	7.18 ± 1.03e	12.79 ± 1.25e	1.48 ± 0.22ab	811.02 ± 6.36g
	30	6.80 ± 1.11e	12.29 ± 1.54g	1.47 ± 0.31ab	1421.04 ± 6.41a

*Note*: Values are given as mean ± SD. *n* = 3. Different letters (a–j) within a column indicate significant difference (*p* < .05). IC50 = The sample concentration providing 50% inhibition.

L*, a*, b*, Δ*E*, hue, and chroma values of IR puffed rice (10 cm distance and 550 W IR power) were 72.56, 1.27, 16.51, 10.98, 16.56, and 1.49, respectively.

It was reported that the popcorn color is related to expansion properties and similar results were observed in popcorn IR popping (Shavandi et al., 2022). The effects of barrel zone's temperature and feed moisture were investigated on extruded whole grain products. The higher temperature and lower moisture produce a better appearance of color (Oliveira et al., 2017). The puffing effect through iron pan on the brown rice color was determined. The puffing shows significant effects on the brown rice color (Mir et al., 2016). Furthermore, it was reported that IR radiation can be used to change the cardamom seeds' color (Shavandi, Kashaninejad, et al., 2020).

### 3.3 TPC analysis

**FIGURE 3:**
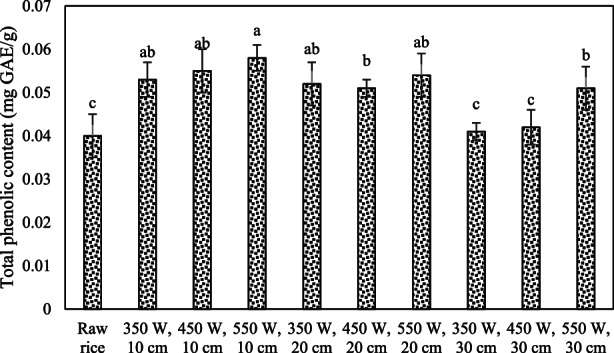
The effect of infrared (IR) on the total phenolic content (TPC) (mg GAE (gallic acid equivalent)/g ± SD) in puffed rice. Different letters (a–c) indicate significant difference (*p* < .05).

The TPC of plants has antioxidant attributes (Lahmass et al., 2017). The IR puffing effect on TPC in puffed rice is shown in Figure 3. The TPC at optimum IR treatment (10 cm distance and 550 W IR power) was 0.058 mg GAE (gallic acid equivalent)/g. The IR puffing treatment effect (increasing the IR power and reducing the distance) on TPC in puffed rice (TPC increased) was significant (*p* < .05).

The popcorn expansion causes the change in carotenoids and TPC (Paraginski et al., 2016). The thermal processing might cause a decrease in TPC. This could be due to oxidation and thermal degradation (Randhir et al., 2008). In a study, it was reported that sand roasting of oats causes a significant decrease in TPC (Gujral et al., 2011).

The iron pan puffing effect on brown rice antioxidant attributes was determined. The TPC at puffing significantly decreased as compared to crude rice (Mir et al., 2016).

Temperature increase may lead to a decrease in TPC (Esmaeelian et al., 2020). In a study, the IR radiation effect on TPC was investigated in paprika powder. The TPC was significantly decreased. The IR irradiation has shown significant effect on the inhibition of polyphenols' degradation as well as the polyphenol oxidase enzyme (Shavandi, Taghdir, et al., 2020).

### 3.4 Antioxidant activity analysis

**FIGURE 4:**
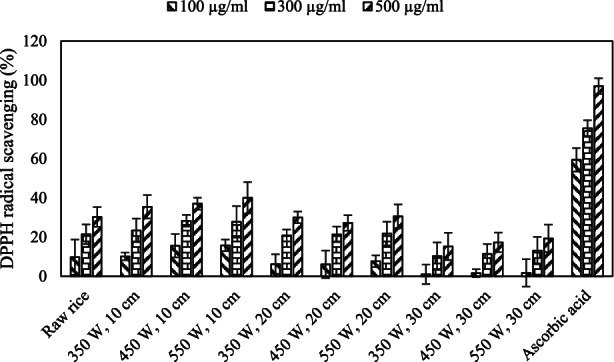
The effect of infrared (IR) on 2,2‐diphenyl‐1‐picrylhydrazyl (DPPH) radical scavenging activity (%) in puffed rice.

The IR effect on DPPH radical scavenging activity is shown in puffed rice and ascorbic acid in concentrations of 100, 300, and 500 μg/ml in Figure 4 and the amount of IC_50_ is shown in Table 2. The DPPH radical scavenging activity in puffed rice was significantly reduced (*p* < .05). The IR effect on IC_50_ at puffed rice was significant (*p* < .05). The IC_50_ value in IR treatment at 10 cm distance and 550 W IR power was obtained at 664.30 ± 5.35. By decreasing the distance and increasing the IR power, more antioxidant activity is maintained.

Chen et al. (2019) reported that antioxidant compounds due to hydrogen‐donating capacity have DPPH radical scavenging attributes. It was reported that due to decrease of process time, the antioxidants experienced lower temperature stress and more of antioxidant compounds were preserved (Esmaeelian et al., 2020). Puffing of puffed rice has shown a significant decrease in antioxidant attributes as compared to crude rice (Mir et al., 2016). The IR popping effect on the DPPH radical scavenging activity in popped popcorn was significant (Shavandi et al., 2022).

### 3.5 Peroxide value

A rise in peroxide value implies primary lipid oxidation, mainly brought about by the formation of hydroperoxide. Peroxide value is also one of the most crucial quality control factors for oil (Feng et al., 2020; Lavanya et al., 2019).

**FIGURE 5:**
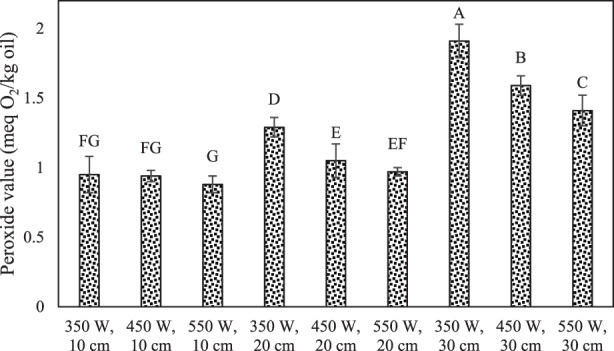
The effect of infrared (IR) on the peroxide value (milliequivalent (meq) O_2_/kg oil) in puffed rice.

The effect of IR on peroxide value in puffed rice is shown in Figure 5. The peroxide value in puffed rice was significantly changed (*p* < .05). The peroxide value at IR treatment using 550 W IR power and 10 cm distance was obtained as 0.88 meq O_2_/kg oil. By increasing the power of lamp and decreasing the sample distance from the IR source, the amount of peroxide value was decreased. This effect is probably due to the increase in process time with decreasing intensity of treatments.

The peroxide value is used to express the oil oxidation level and hydroperoxides start autoxidation of oil rancidity through oxygen uptake and subsequently produce carbonyl compound by‐products (Park et al., 2002). The fatty acids' oxidation leads to the production of peroxides. The peroxides can show an adverse effect on quality and the health of oils, and also on foods containing these oils. The storage and processing temperatures and puffed corn porosity are the main factors in increasing oxidation of oil (Hashempour‐Baltork et al., 2018).

### 3.6 SEM analysis

The scanning electron microscopy (SEM) analysis was used to determine the surface structural change in puffed rice through IR. The surface structural change in puffed rice in different treatments of IR puffing is shown in Figure 5.

**FIGURE 6:**
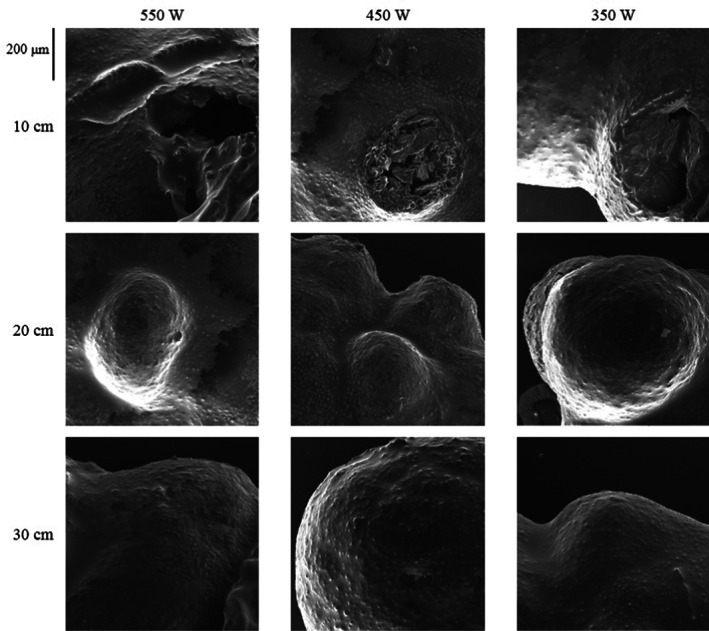
Scanning electron microscopy (SEM) of puffed rice at different treatments of puffing through infrared (IR).

In Figure 6, numerous protrusions were observed on the surface of the samples. By decreasing the sample distance from the IR lamp and increasing the IR lamp power, the size of protrusions was increased (the volume of the protrusions). The maximum increase in the protrusions size was observed in 10 cm distance and 550 W IR power. According to Figure 6 on 550 W IR power and 10 cm distance, the increase in volume occurred rapidly and led to rupture of the protrusion. This effect has been observed more gently in 10 cm distance and 450 W IR power, and 350 W IR power and 10 cm distance treatments.

Increase in the protrusions size means an increase in the volume of puffed rice and shows the process efficiency in the puffed rice. The results of the IR puffing attributes of puffed rice as shown in Table 1 correspond to the SEM in Figure 6. By increasing volume puffing, the protrusions size was increased.

The popcorn palatability and softness may be related to a higher volume of expansion (Dofing et al., 1990). In a study, it was reported that the popcorn texture is related to expansion properties (Ceylan & Karababa, 2002). The gun puffing effect on common wheat, emmer wheat, rice, rye, barley, and buckwheat was determined. In SEM, the puffing process causes significant change in structure and the physical attributes of sample which were related to our results (Mariotti et al., 2006).

### 3.7 FTIR spectroscopy

**FIGURE 7:**
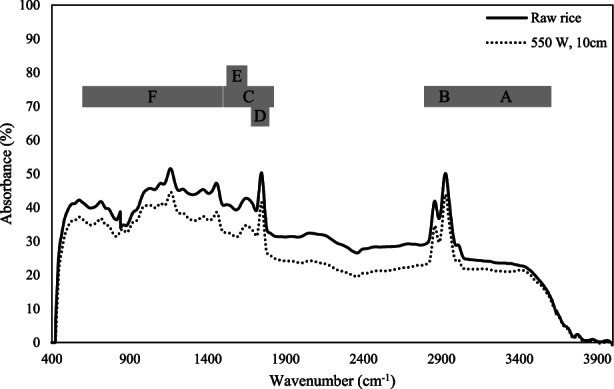
The effect of infrared (IR) expansion on the bioactive component of puffed rice by Fourier transform infrared (FTIR) spectroscopy. Different letters (A–F) are indicated in Table 3.

The IR puffing effect in optimum treatment of IR puffing (10 cm distance and 550 W IR power) on the food component of puffed rice and raw rice was determined. Figure 7 shows FTIR spectroscopy of food components in puffed rice and raw rice. The adsorption range of functional groups at food compounds is shown in Table 3 and the food compounds' absorption peaks of puffed rice are determined. The IR puffing effect on food compounds of puffed rice compared to raw rice was significant (*p* < .05).

**TABLE 3:** The range of adsorption of functional groups in bioactive compounds

Group	Component	Peak (cm^−1^)	The characteristic in Figure 7	References
O–H	Carbohydrates or amide structures as well as moisture	From 3600 to 3025	A	Forato et al. (1998); Naumann et al. (2010)
C–H	Symmetric and asymmetric stretching vibrations of methyl and methylene groups	From 3025 to 2790	B	Guillen and Cabo (1997); Kim et al. (2007)
C=O	Amide bands	From 1770 to 1500	C	Duodu et al. (2001); Ismail et al. (1993); Kim et al. (2007); Movasaghi et al. (2008); Naumann et al. (2010)
	Fatty acid esters Free fatty acids	1743 and 1709	D	
	Secondary structure of proteins	1643 and 1539	E	
The fingerprint region	Mostly related to carbohydrate structures	Around 1500 to 600	F	Kuhnen et al. (2010)

According to Figure 7, the IR puffing effect on food compounds of puffed rice such as mostly related to the carbohydrate structure (Figure 7, F), the secondary structure of protein (Figure 7, E), free fatty acids and fatty acid esters (Figure 7, D), amide bands (Figure 7, C), and asymmetric and symmetric stretching vibrations of methylene and methyl group (Figure 7, B), relative to raw rice, was significant (*p* < .05).

The C–H stretching vibrations are visible in the range of 3025 to 2790 cm^−1^ with absorption bands at 3006, 2924, and 2853 cm^−1^ that are brought about by symmetric and asymmetric stretching vibrations of the methyl and methylene groups, respectively (Guillen & Cabo, 1997; Kim et al., 2007). The signals in the range of 1770 to 1500 cm^−1^ are bands of amide vibration and C=O stretching. While the wider bands at 1643 and 1539 cm^−1^ are related to the secondary structure of proteins, absorption bands at 1743 and 1709 cm^−1^ can be assigned to the C=O stretching of fatty acid esters and free fatty acids, respectively (Duodu et al., 2001; Ismail et al., 1993; Kim et al., 2007; Movasaghi et al., 2008; Naumann et al., 2010; Sow & Yang, 2015). There are multiple overlapping bands in the fingerprint area (1500–600 cm^−1^), most of which are associated with carbohydrate compounds (Kuhnen et al., 2010).

Fourier transform infrared spectroscopy is a fast method and nondestructive type that can be used to identify attributes (Achten et al., 2019; Yang et al., 2018). The IR popping effect on the food compounds of popped popcorn was investigated. The IR popping can change the popped popcorn food compounds (Shavandi et al., 2022). Based on the results, IR puffing could be changing the puffed rice food compounds.

## 4 CONCLUSION

In this study, the puffing process effect on some physicochemical properties (antioxidant activity, TPC, color, peroxide value, SEM, and FTIR) of puffed rice (*Oryza sativa* L.) was investigated. The puffing process exhibited a significant effect on physicochemical properties of puffed rice. In color index, just change of b* in IR puffed rice was significant (*p* < .05). Δ*E* and hue were significantly changed in IR puffed rice (*p* < .05). Chroma were nonsignificantly changed in IR puffed rice (*p* > .05). In the SEM analysis, an increase in the protrusions size of puffed rice means an increase in the volume of samples and shows the efficiency of puffing in puffed rice (with increasing the power of the lamp and decreasing the sample distance from the IR lamp, the protrusions size was increased). According to the FTIR, IR puffing could be changing the puffed rice food compounds. The TPC and DPPH radical scavenging activity in puffed rice were significantly reduced (*p* < .05) during the puffing of IR. According to the puffing attributes of puffed rice, the optimum treatment for the IR puffing of puffed rice were 10 cm distance and 550 W IR power. IR puffing is a high efficiency method of puffing. Finally, it can be suggested that the IR puffing method can be investigated for the puffing of cereals and grains.

## CONFLICT OF INTEREST

All authors have no conflict of interest to report.

## Data Availability

The data that support the findings of this study are available on request from the corresponding author. The data are not publicly available due to privacy or ethical restrictions.
